# Antioxidant and Anti-Inflammatory Properties of Walnut Constituents: Focus on Personalized Cancer Prevention and the Microbiome

**DOI:** 10.3390/antiox12050982

**Published:** 2023-04-22

**Authors:** Nuoxi Fan, Jennifer L. Fusco, Daniel W. Rosenberg

**Affiliations:** Center for Molecular Oncology, University of Connecticut Health Center, Farmington, CT 06030-3101, USA

**Keywords:** cancer prevention, walnut, inflammation, microbiome, gluten-free, ellagitannins

## Abstract

Walnuts have been lauded as a ‘superfood’, containing a remarkable array of natural constituents that may have additive and/or synergistic properties that contribute to reduced cancer risk. Walnuts are a rich source of polyunsaturated fatty acids (PUFAs: alpha-linolenic acid, ALA), tocopherols, antioxidant polyphenols (including ellagitannins), and prebiotics, including fiber (2 g/oz). There is a growing body of evidence that walnuts may contribute in a positive way to the gut microbiome, having a prebiotic potential that promotes the growth of beneficial bacteria. Studies supporting this microbiome-modifying potential include both preclinical cancer models as well as several promising human clinical trials. Mediated both directly and indirectly via its actions on the microbiome, many of the beneficial properties of walnuts are related to a range of anti-inflammatory properties, including powerful effects on the immune system. Among the most potent constituents of walnuts are the ellagitannins, primarily pedunculagin. After ingestion, the ellagitannins are hydrolyzed at low pH to release ellagic acid (EA), a non-flavonoid polyphenolic that is subsequently metabolized by the microbiota to the bioactive urolithins (hydroxydibenzo[b,d]pyran-6-ones). Several urolithins, including urolithin A, reportedly have potent anti-inflammatory properties. These properties of walnuts provide the rationale for including this tree nut as part of a healthy diet for reducing overall disease risk, including colorectal cancer. This review considers the latest information regarding the potential anti-cancer and antioxidant properties of walnuts and how they may be incorporated into the diet to provide additional health benefits.

## 1. Introduction

Walnuts (*Juglans regia* L.) are a key part of the Mediterranean diet. Walnut consumption has been associated with many health benefits, including reduced cardiovascular disease (CVD), coronary heart disease, and type II diabetes, as well as beneficial effects on aging and cancer risk [1]. Liu et al. [2] recently reviewed the association of walnuts with mortality and life expectancy and concluded that higher walnut consumption was associated with lower risks of total and CVD mortality and with greater life expectancy among an elderly U.S. population. To examine potential associations of walnuts with cardiac structure and function in the CARDIA study, Steffen et al. [3] reported that reduced coronary artery risk in young- to middle-aged adults was associated with improved diastolic dysfunction associated with walnuts. Sabate, Ros and colleagues [4] evaluated whether dietary walnuts may improve lipid profiles independently of geography or dietary history. Data from their WAHA (Walnuts and Healthy Aging) two-year randomized controlled trial showed that daily walnut inclusion in the diet of elderly individuals resulted in modest LDL-C reduction [4]. The data also revealed beneficial effects of walnut on lipoproteins, with reductions in intermediate-density lipoprotein cholesterol and total LDL [4].

Walnuts, in comparison to other tree nuts, such as almonds, pecans and hazelnuts, contain the highest content of plant-based polyunsaturated fatty acids (PUFAs), including ALA [5]. The incorporation of 1 oz of nuts, including walnuts, 5 times a week into a healthy diet has been recommended by the Dietary Guidelines for Americans 2020–2025 [6]. According to the USDA, walnuts provide both protein and fiber and are the only nut source that is an excellent source of omega-3 ALA (2.5 g/oz) [6,7]. Walnuts are an extremely versatile food and can be added to a variety of food preparations. They are also a plant-based alternative to meat products and pair well with other nutrient-high foods.

In several preclinical animal studies, reduced tumor volume/multiplicity, altered gene expression and increased microbiome diversity have all been observed following walnut/nut consumption. In human studies, which include clinical trials and cohort studies, increased walnut/nut intake leads to an overall decreased cancer risk and increased microbiome diversity. Mechanistic studies in cell culture systems have uncovered altered gene expression profiles and anti-inflammatory changes when cells are treated with walnut phenolic extract (WPE) or other walnut constituents. Examples of each of these effects will be discussed in the following sections. These properties of walnuts provide the rationale for its inclusion into a healthy diet that may reduce overall disease risk, including diseases of the gastrointestinal (GI) tract.

In view of the potential anti-cancer properties of walnut and its individual constituents, this review aims to provide a comprehensive overview of the field. A particular emphasis is placed on the anti-inflammatory properties of the walnut and the potential modulation of the gut microbiome as related to disease risk. This review summarizes our current knowledge base, focusing on clinical studies, as well as preclinical models and cell culture systems.

## 2. Walnut Constituents

**Figure 1:**
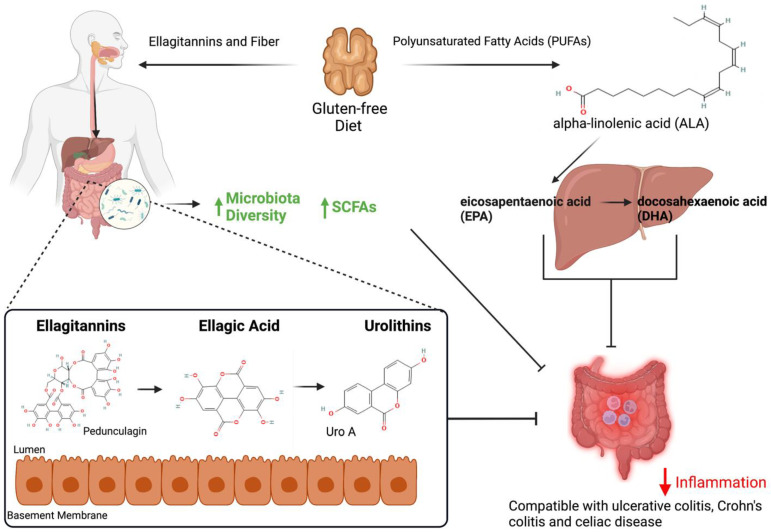
Walnuts are a rich source of antioxidant polyphenols (ellagitannins (ETs), fiber, and polyunsaturated fatty acids (PUFAs)) as well as being gluten-free in their natural state. Upon ingestion, ETs are metabolized to ellagic acid (EA) under low pH, and then further metabolized to urolithins by the intestinal microbiota within the lumen. Walnuts contain PUFAs, particularly alpha-linolenic acid (ALA), which is metabolized to eicosapentaenoic acid (EPA) and docosahexaenoic acid (DHA) in the liver. Fiber increases microbiota diversity which contributes to the conversion of fiber into short chain fatty acids (SCFAs). Walnuts are also naturally gluten-free and once absorbed, ETs, ALA and SCFAs increase anti-inflammatory activity within the intestinal lining that may help counter diseases such as ulcerative colitis, Crohn’s colitis, and celiac disease as well as having possible anti-cancerous effects. Created with BioRender.com. Compound structures are from PubChem.

Walnuts contain a remarkable array of micronutrients and phytochemicals that exhibit a wide range of health benefits, including protection against inflammation and various forms of cancer. Walnuts are a rich source of polyunsaturated fatty acids (PUFAs), tocopherols, antioxidant polyphenols (catechins, resveratrol, ellagitannins), phytosterols (stigmasterol, campesterol, sitosterol) and active prebiotics [8,9,10,11,12,13] (Figure 1). Moreover, walnuts are a rich source of fiber that generates SCFAs, metabolic products that can reduce cancer risk [14,15]. Among the tree nuts, walnuts contain the most favorable ratio of PUFAs (e.g., α-linolenic acid, ALA) to saturated fatty acids at an average ratio of 8.2:1, depending on the source and variety of walnuts [5,7,16]. However, the health benefits of walnuts should not be attributed to a single component, but more likely result from interactions among multiple compounds present within the nut [1]. While acknowledging this rich array of nutrients present within the walnut, this review will focus primarily on the health benefits of the PUFAs and the polyphenols.

### 2.1. Alpha-Linolenic Acid

Walnuts contain large amounts of ALA (2.5 g/oz) that humans must obtain from dietary sources. Humans lack the complement of enzymes required for the de novo synthesis of ALA [10]. ALA is an important metabolic precursor to eicosapentaenoic acid (EPA, 20:5) and docosahexaenoic acid (DHA, 22:6) and is converted to EPA and DHA in the liver [11] (Figure 1). In males, the conversion of ALA to EPA and DHA is 8% and 4%, respectively [17], whereas in females, 21% of ALA is converted to EPA and 9% to DHA, indicating gender specificity [18]. These lipid metabolites play a key role in the subsequent formation of an array of anti-inflammatory products, including the protectins and resolvins, a group of specialized pro-resolving mediators (SPMs) with well-documented effects on immune and inflammatory cells [11,19,20,21].

ALA (and omega-3 PUFAs) have been shown to exhibit anti-inflammatory activity and tumor suppressive properties in animal models and cell culture systems [19,22,23,24], although its direct association with cancer risk is debated [25,26,27,28,29]. Using a high-fat-diet-induced type 2 diabetes model, ALA prevented NLRP3 inflammasome-dependent inflammation [21]. ALA was tested in HT-29 CRC cells and showed concentration-specific effects on cell viability and apoptosis-related cell death [30]. ALA was also shown to suppress osteosarcoma cell proliferation and metastases by inhibiting fatty acid synthase [31]. In triple-negative breast cancer cells, ALA reduced Twist1 protein levels and cell migration, while inhibiting protein expression of key mesenchymal markers [32]. With MCF-7 and B16-BL6 cancer cells, flaxseed oil containing ALA, DHA and EPA inhibited cancer cell growth and induced apoptosis [33]. ALA provided protection to mice in a carcinogen-induced breast cancer model, while activating mitochondrial apoptosis and inhibiting fatty acid synthesis in ER+MCF-7 breast cancer cells [34]. Finally, Todorov et al. [20] maintained mice on an ALA-rich diet (20% perilla oil) and showed that ALA increased EPA and DHA levels, while reducing arachidonic acid (AA) in the intestine [20]. The diet also caused morphologic changes to the intestine, but these mucosal changes were unlikely to be related specifically to ALA [20].

### 2.2. Polyphenols

Phenolic compounds are important micronutrients that are abundant in walnuts [8,9]. Polyphenolic compounds (flavonoids and non-flavonoids), although generally poorly absorbed, have a wide range of anti-bacterial, anti-inflammatory and anti-carcinogenic effects [35]. Based on serving size, walnuts are the seventh largest source of total polyphenols among commonly consumed foods and beverages [8,36]. As comprehensively reviewed by Lipinska et al. [37], ellagitannins belong to the class of compounds referred to as hydrolyzable tannins. As such, they are esters of hexahydroxydiphenoic acid and monosaccharide and can be hydrolyzed in the digestive tract to release the polyphenol, ellagic acid [37]. Among the various tree nuts, walnuts are enriched in the ellagitannin, pedunculagin, with levels reaching approximately 16 mg/g [38].

The phenolic profiles and antioxidant activities of free, esterified and bound phenolics in the walnut kernel reveal the presence of a remarkable array of phenolic compounds, including phenolic acids, flavonoids, tannins, phenolic lignans and stilbene derivatives [39]. The main polyphenol found in walnuts is pedunculagin, an ellagitannin that has a wide range of antioxidant and anti-inflammatory properties [8]. After ingestion, ellagitannins are hydrolyzed to release EA, which is converted by the gut microflora into the urolithins [8]; this aspect of polyphenol metabolism and their chemopreventive properties are discussed in detail later in this review.

## 3. Methods

### Search Strategy and Study Selection

A comprehensive search for eligible articles was conducted using Pubmed/MEDLINE. Multiple search terms were combined to focus the search. The keywords or search terms included “cancer” OR “tumor” OR “walnuts” OR “nuts” OR “urolithins” OR “gut health” OR “inflammation” OR “diet” OR “PUFA” OR “fiber” OR “alpha-linolenic acid (ALA)” AND “colon” OR “colorectal”. These search terms were used in variable order and combinations. Filters (including human, mouse, rat, and cell culture, and English language text) were used to narrow down search results. Databases were searched from 2002–2022. This period was chosen to reflect the most current studies conducted and published. One article was searched using the URL for validation and the full article was provided to us by the author. Studies were downloaded to EndNote 20 citation management software and duplicates were deleted prior to screening. All reviewers screened articles independently by the title and abstract first and then screened using the full text to determine eligibility. There was no limitation on the country of publication, but non-English articles were excluded from this narrative review. Articles that had no accessible full text were also not included. Articles that did not involve walnuts and walnut constituents were also excluded. There were no limitations concerning age, education, ethnicity, or socioeconomic status within the human studies and no limitation concerning type of cancer, age, genotype and cell lines within the animal and cell culture studies.

## 4. How Do Walnuts Influence Cancer Risk?

### 4.1. Animal Studies

Preclinical studies have examined the effects of walnut on cancer development (Table 1). In an early study, Hardman and Ion [40] tested whether walnut consumption affects growth of human breast cancer cells implanted into nude mice. The study tested efficacy after tumors had reached 3–5 mm diameter at a human walnut concentration equivalent to 56 g/day. Tumor growth and cell proliferation were significantly reduced [40]. Walnuts were then shown to reduce tumor incidence, multiplicity, and size in a mouse mammary gland primary tumor model [41]. Using a targeted expression array of 84 genes, walnuts were found to alter key signaling pathways involved in proliferation, cell differentiation and apoptosis. Davis and colleagues [42] tested a high-fat diet containing whole walnuts on prostate cancer growth in TRAMP mice. A high-fat diet (20% of energy) with 155 g of whole walnut was compared to a micronutrient-matched diet with soybean oil providing either 20% (high-fat) or 8% (low-fat) of total energy. Prostate tumor weight and growth were reduced by walnuts, accompanied by a decline in plasma IGF-1, resistin and LDL [42]. The authors concluded that the walnut effect is not based entirely on fatty acid or tocopherol content, emphasizing the importance of whole foods rather than individual constituents.

Mantzoros and colleagues [43] evaluated the effects of walnut in a tumor explant model using human HT-29 CRC cells. Tumor growth was determined in walnut- and flaxseed-fed mice compared to corn oil controls. While no differences were observed in metabolic and hormonal profiles, or in serum antioxidant capacity, both walnut and flaxseed oil caused a significant decrease in angiogenesis, assessed by CD34 that was absent in the flaxseed oil group, while the area of cellular necrosis within the tumor was doubled [43]. Tsoukas et al. [44] examined potential effects of walnut on microRNA expression in an HT-29 tumor explant study. Seven days after tumor cell injection, mice were randomized to either a control or walnut-containing diet for 25 days. Walnuts caused significant elevation in tumor tissue levels of ALA, EPA and DHA, while reducing AA levels. Consistent with the known effects of omega-3 FA, tumor size was negatively correlated with total omega-3 FA composition.

Nakanishi et al. assessed potential benefits of walnut in a mouse tumor bioassay using the potent colon carcinogen, azoxymethane (AOM) [45]. Results showed a modest reduction in colon tumors in mice fed a standard laboratory diet (AIN-76A) containing 9.4% walnuts (15% of total dietary fat). In a second study, walnuts were added to a Total Western Diet (TWD [45,46,47]) designed to represent the median values of micro- and macro-nutrients consumed by a significant proportion of Americans. There was a significant reduction (2.3-fold) in tumor numbers in male mice fed TWD containing 7% walnuts (10.5% of total fat). Since mice fed with higher concentrations of walnuts lacked cancer protection, particularly in females, it was postulated that optimal levels of dietary walnut may be most effective for long-term cancer prevention [45]. Obesity associated with visceral fat is a risk factor for CRC and contributes to elevated cancer incidence in mice [48]. Together with the Huffman laboratory [49], we evaluated whether walnuts may provide protection against obesity-related elevation in cancer risk in *Apc^1638N/+^* and *Apc^Δ14^* mice by focusing on colon stem cell dynamics. *Apc^1638N/+^* mice were fed either a low-fat diet (LFD) or a high-fat diet (HFD), with or without walnuts. After 24 weeks, mice fed HFD supplemented with walnuts had a reduced (32%) tumor burden compared to HFD alone. Histological assessment revealed a reduction in tubulovillous-type adenomas and adenocarcinomas in HFD-fed mice supplemented with walnut. Notably, walnut consumption reduced circulating pro-inflammatory CCL5, while maintaining stem cell function under HFD-induced stress [49].

**Table 1:** Summary of animal studies (*n* = 10) in a narrative review that focuses on walnut-induced changes in preclinical models and cancer cell lines.

Author(s), Year, Reference	Animal Model(s)	Experimental Design	Key Findings
Hardman et al., 2011 [41]	C3(1) TAg murine breast cancer model	Maternal diet: Corn oil vs. walnut diet Offspring diet: Corn oil/Corn oil; Corn oil/Walnut Walnut/Walnut; Walnut/Corn Oil Walnut-containing diets were 18% of total calories	Decreased tumor size, multiplicity and incidence with walnut diet Walnut consumption linked to altered expression of several genes connecting to mammary epithelial cell differentiation and proliferation
Koh et al., 2019 [50]	Acute (DSS) colitis; chronic colitis (IL-10-KO) and inflammation-associated CRC (AOM + DSS) mouse models	Walnut Phenolic Extract (WPE) (20 mg/kg) via oral gavage for 2 weeks Acute colitis: C57BL/6 mice given 4% DSS for 5 days Chronic colitis: IL-10 (-/-) mice Inflammation-cancer model: AOM + 2% DSS × 3 cycles	Reduced disease severity in both acute and chronic colitis models, including the IL-10 -/- mice
Hardman and Ion, 2008 [40]	Athymic nude mice (*nu/nu*) + MDA-MB 231 human breast cancer cells	After tumors reached 3–5 mm in diameter, mice were placed on the following diets: Corn oil (control) vs. Walnut diet (18% of total calories)	Overall decrease in tumor cell proliferation Increase in liver EPA and DHA levels
Guan et al., 2018 [49]	Intestinal tumorigenesis in *Apc^1638N/+^* and *Apc^D14/+^* mice MC38 CRC explant Obesity/high calorie diet	*Apc^1638N/+^*: 1. Control low-fat diet (LFD) (*n* = 11) 2. LFD + walnuts (6% by weight) (*n* = 12) 3. Control high-fat diet (HFD) (*n* = 18) 4. HFD + walnuts (7.7% by weight) (*n* = 23) *Apc^Δ14^* 1. The western diet (TWD) + 0% walnuts (males *n* = 12, females *n* = 10) 2. TWD +7% walnuts (males *n* = 11, females *n* = 9) C57BL/6J male (*n* = 48) 1. LFD (*n* = 12) 2. LFD + walnuts (*n* = 12) 3. HFD (*n* = 12) 4. HFD + walnuts (*n* = 12)	Reduction in CCL5 levels Preservation of intestinal stem cell function under conditions of HFD Reduced tumor multiplicity
Nakanishi et al., 2016 [45]	A/J—AOM mouse CRC model	AIN-76A diet (4 weeks old) 0%, 9.4%, 14.1%, or 18.8% of walnuts by weight TWD (Total western diet) (4 weeks old) 0% 3.5%, 7%, or 14% of walnut by weight Six weekly injections of AOM *(i.p.*) at 5 weeks of age.	Reduction in tumor number in AIN-76A diet with 9.4% walnut Tumor protection more pronounced in males at 7% walnut Increase in bacterial richness and diversity
Tsoukas et al., 2015 [44]	HT-29 CRC explant model in athymic nude (*nu/nu*) mice	Control diet (*n* = 16) AIN-79 + corn oil (100 mg/kg) Walnut diet (*n* = 16) AIN-79 + Walnut (18.8% of calories) + Corn oil (100 mg/kg)	Increase in alpha-linolenic acid (ALA), eicosapentaenoic acid (EPA), docosahexaenoic acid (DHA) and total omega-3 acids Decrease in arachidonic acid Decrease in tumor growth rate and miRNA expression in walnut group
Davis et al., 2012 [42]	High-fat diet in TRAMP mouse model for prostate cancer	Mice are placed on diets at 8 weeks of age AIN-93M + whole walnut 20.5% of energy from fat AIN-93M + soybean oil (HFD) 100 g/kg) 20.7 % of energy from fat AIN-93M + soybean oil (LFD) 40 g/100 kg) 8.7% of energy from fat Mice were sacrificed at 9, 18 and 24 weeks after feeding	Reduction in prostate tumor weight and growth rate in whole walnut diet Decrease in LDL, resistin, plasma insulin-like growth factor 1, and prostate weight in whole walnut diet at 18 weeks Difference in liver metabolites at 9 and 18 weeks post- feeding in groups
Nagel et al., 2012 [43]	HT-29 CRC explant model in athymic nude (*nu/nu*) mice	Mice were placed on diets at 7 weeks old (*n* = 16/group) Corn oil diet (control) AIN-76 Walnut diet 18.8% of a 2000 calorie per day diet Flaxseed oil diet 18.8% of a 2000 calorie per day diet	Tumor growth rate decreased in walnut and flaxseed fed groups Walnut- and flaxseed-fed groups exhibited decreased expressions levels of angiogenesis factors in serum
Chen et al., 2020 [51]	A/J—AOM mouse CRC model	Six weekly injections of AOM (*i.p.*) at 5 weeks old; control mice were injected with vehicle (0.9% NaCl) AOM group diets (*n* = 20/group, 10 males, 10 females) 1. 0% walnut level 2. 3.5% walnut level 3. 7% walnut level 4. 14% walnut level Control group diets (*n* = 10/group, 5 males, 5 females) 1. 0% walnut level 2. 7% walnut level Fecal samples collected at 6, 11, 13, 16, and 20 weeks of age.	3 enterotype-like clusters identified (E1, E2, E3) E1 and E3 were enriched with *Bacteriodaceae*, driven by different operational taxonomic units E2 were enriched with *Porphyromonadaceae* and *Lachnospiraceae* E2 looks to represent the early stage of the microbial status in the mice
Byerley et al., 2017 [52]	Fischer 344 rats	Walnut group (*n* = 10) 11% by weight ground walnuts Replacement group (*n* = 10) corn oil, alphacel fiber, and casein added to replace walnut Fecal samples collected at sacrifice	Rats on walnut diet showed greater microbial species diversity Increased amount of *Firmicutes* and reduced amount of *Bacteriodetes* Microbiome enriched with probiotic-like bacteria (e.g., *Lactobacillu*, *Ruminococcaceae*, and *Roseburia)*; reduction in *Bacteriodes* and *Anaerotruncus*

### 4.2. Human Studies

Several promising clinical studies and meta-analyses focused on cancer biomarkers have been conducted using tree nuts and walnuts (Table 2). In a case-control study of peanuts, walnuts, and almonds in Mexican-mestizo subjects, Soriano-Hernandez et al. [53] reported that high consumption of these foods was associated with a 2- to 3-fold reduction in breast cancers. In a pilot two-arm clinical trial of 10 subjects, Hardman et al. [54] examined how dietary walnuts may alter gene expression related to tumor growth, survival, and metastasis in breast cancer patients. They hypothesized that walnut consumption alters gene expression in breast cancer samples in a manner consistent with their earlier preclinical findings in mice [40]. RNAseq showed 456 genes that were altered by walnut in surgical specimens after two weeks of walnut treatment. Pathway analysis identified activation of signaling pathways that promote apoptosis and cell adhesion, and down-regulation of cell proliferation and migration pathways [40,55]. Supporting mRNA expression data was published subsequently in an accompanying report [55], providing detailed analysis of gene networks.

As part of the Goletan Cohort Study, peanuts, walnuts, and mixed nut intake was assessed in 50,045 participants in Northeastern Iran (2004–2008) using a validated food frequency questionnaire [56]. The goal was to investigate associations between nut consumption and risk of esophageal squamous cell carcinoma (ESCC) in an area with high incidence of this disease. The authors found an inverse association between total nut consumption and risk of ESCC in this cohort, although several study limitations were acknowledged, including the influence of preparation methods, an important factor that is not often considered [56]. A recent meta-analysis [57] of total nut, tree nut and peanut consumption and cancer incidence and mortality reported a 4% reduction in cancer mortality with higher nut intake, consistent with a recent meta-analysis by Zhang et al. [58] However, other studies from the Giovannucci group [59,60] have shown limited efficacy of tree nut consumption and cancer risk. In a prospective study of nut consumption and risk of primary hepatocellular carcinoma (HCC), Sui et al. [59] examined data collected in the NHS and HPFS and reported that nut consumption (walnuts and peanuts) was not strongly associated with HCC risk. Additionally, Fang et al. [60] analyzed data from the same prospective cohorts for health effects of various nuts, including walnuts. In contrast to earlier meta-analyses and case-control studies, no significant association was observed with the risk of total cancers, including lung, colorectal, bladder, breast, and prostate cancer [60]. The authors note that their study rigorously controlled for established or suspected risk factors. Clearly, as new clinical studies are conducted world-wide, follow-up analyses and updates will be critical for establishing dietary recommendations.

In a longitudinal, observational cohort study, Fadelu et al. [61] found that consumption of walnuts (and other nuts) was associated with reduced CRC risk. Patients with stage III CRC were enrolled into a randomized adjuvant chemotherapy trial designed to analyze associations between dietary intake and incidence of cancer recurrence and death using dietary intake reports and food frequency questionnaires. It was found that patients consuming two or more servings of nuts each week, particularly tree nuts, showed significant improvement in both disease-free and overall survival [61]. This exciting finding provides the impetus for understanding the mechanisms underlying improved patient outcome.

Our laboratory recently conducted a clinical study involving walnut consumption in 39 healthy subjects [62]. Urine samples were collected before and after three weeks of walnut supplementation (56 g/day). Nine urolithin metabolites were measured in urine by UPLC-MS/MS, before and after walnuts. To optimize the analytical method, including determining metabolite stability in the biospecimens, a pilot study was conducted on urine samples from the first 10 patients. As reported [62], there was a remarkable inter-individual variability in urolithin levels, a finding that is consistent with earlier studies [63]. This analytical approach will be readily applied to future studies of urolithin metabolism.

As reviewed by Marafini et al. [64], a small, but significant increased risk of cancer has also been found in patients that suffer from celiac disease (CD), a chronic enteropathy developing in susceptible individuals after ingestion of gluten [64]. These CD-associated neoplasias involve mainly the gastrointestinal (GI) tract, including small bowel adenocarcinoma and enteropathy-associated T-cell lymphoma (EATL) [64]. A hallmark of CD is its association with a marked imbalance in the overall systemic oxidant/antioxidant balance, particularly within the GI tract. Since CD is a life-long condition with long-term consequences, the inclusion of gluten-free foods into the diet that contain copious quantities of natural plant-based antioxidants might prove beneficial in the long-term management of this disease and its associated cancer risk. Whole walnuts are gluten-free and known to increase dietary fiber [65,66], nutritional qualities that would certainly be beneficial to CD patients. Furthermore, high fiber diets increase microbial diversity, which may help reduce the inflammatory response and promote the growth of beneficial bacteria in the gut [67]. Thus, considering the remarkable array of nutrients present in the walnut, it is likely that adding this tree nut to a gluten-free diet may offer a safe and effective strategy for reducing oxidative stress and cancer risk associated with CD.

Overall, the preclinical and clinical studies described herein provide further support for the anti-cancer properties of tree nuts. A summary of intermediate biomarkers associated with nut consumption may be useful for tracking efficacy in future clinical trials.

**Table 2:** Summary of human studies (*n* = 12) in a narrative review on the effects of nut intake on cancer risk and microbiome.

Author(s), Year, Reference	Patient Population	Experimental Design	Key Findings
Hardman et al., 2019 [54]	Female breast cancer patients (*n* = 10) 18–90 years-old	Data/sample collected Core needle biopsies 5-mL blood collected after diet Diets Walnut group (2 oz/day) until surgery Control group (no walnut)	Expression of 456 identified genes had a significant change in tumors from the walnut diet Inhibition of signaling pathways that promote cell proliferation
Soriano-Hernandez et al., 2015 [53]	Female breast cancer patients (*n* = 97) Healthy control patients (BI-RADS score 1–2) (*n* = 104)	Data/sample collected FFQ	Patients who consumed the largest quantity of peanuts, walnuts or almonds reduced their risk of breast cancer by ~2- to 3-fold
Hashemian et al., 2018 [56]	Healthy subjects (*n* = 50,045) Males and females >40 years-old All subjects recruited from Northeastern Iran (2004–2008)	Data/sample collected FFQ Dietary recall questionnaire	Highest tertile of nut consumption correlated with decreased risk of esophageal squamous cell carcinoma (ESCC)
Sui et al., 2019 [59]	Nurses’ Health Study, Healthy female nurses (*n* = 121,700) 30–55 years old Health Professionals Follow-up Study Healthy males (*n* = 51,529) 40–75 years old	Data/sample collected FFQ	Higher total nut intake showed non-significant association with hepatocellular carcinoma (HCC) risk Higher tree nut intake was associated with lower HCC risk
Fang et al., 2021 [60]	Nurses’ Health Study Healthy females (*n* = 121,700) 30–55 years-old Nurses’ Health Study II Healthy females (*n* = 116,429) 25–42 years-old Health Professionals Follow-up Study Healthy males (*n* = 51,529) 40–75 years-old	Data/sample collected FFQ collected every 2 years Dietary intake questionnaire given every 4 years	Nut consumption was not associated with overall risk of cancer in these cohorts
Fadelu et al., 2018 [61]	Subjects diagnosed with stage III colon cancer (*n* = 826) Males and females	Data/sample collected FFQ	Increased nut intake correlates with reduced cancer recurrence and mortality
Provatas et al., 2021 [62]	Healthy subjects (*n* = 12) Males and females 50–65 years-old	Data/sample collected Urine samples before and after 3-week walnut consumption Diet Three-weeks study design using walnut diet (2 oz/day)	Urolithin levels are increased in study subjects by walnut intake, but levels are highly variable
Bamberger et al., 2018 [12]	Healthy subjects (*n* = 135) Males and females >50 years old	Data/sample collected Stool collection at the end of each diet phase Diet Four-week cross-over design using a nut-free western control diet, (50% carbohydrates, 35% fat, and 15% protein) and walnut diet (43 g/day)	Significant increase in *Ruminococcaceae* and *Bifidobacteria* following walnut consumption Significant decrease in *Clostridium* sp. and *XIVa* species during following walnut consumption Walnut consumption increased microbiome diversity, including enhancement of butyrate-producing species
Bamberger et al., 2017 [68]	Healthy subjects (*n* = 194) Males and females >50 years-old	Data/sample collected Blood sample collected at the end of each diet phase Diet Four-weeks cross-over design using a nut-free western control diet (50% carbohydrate, 35% fat, and 15% protein) and walnut diet (43g/day)	Walnut consumption reduced fasting cholesterol, non-HDL cholesterol, LDL cholesterol, triglycerides and ApoB levels
Holscher et al., 2018 [66]	Healthy subjects (*n* = 18) Males and females 25–75 years-old	Data/sample collected Fecal samples collected before and after each diet phase Blood samples collected before and after each diet phase Diet Three weeks cross over design using nut-free control diet and walnut diet (42g/day)	Walnut consumption increased abundance of *Faecalibacterium*, *Clostridium*, *Dialister*, and *Roseburia* and reduced the abundance of *Ruminococcus*, *Dorea*, *Oscillospira*, and *Bifidobacterium* Lower serum LDL cholesterol after walnut consumption Lower secondary bile salts, deoxycholic acid and lithocholic acid after walnut consumption
Garcia-Mantrana et al., 2019 [69]	Healthy subjects (*n* = 27) >18 years of age Males and females	Data/sample collected Fecal samples Urine samples Diet 3-day study design using walnut diet (33 g/day)Groups Subjects were placed in one of three groups: Metabotype A (UM-A)—Predominantly urolithin A producer Metabotype B (UM-B)—A Urolithin B, iso-urolithin A and urolithin A producers Metabotype 0 (UM-0)—no urolithins producer	UM-B subjects showed increase in in abundance of *Blautia*, *Bifidobacterium* and *Gordonibacter* Both UM-A and -B subjects showed increase in *Coprococcus* and *Collinsella* Increase in short-chain fatty acids (SCFAs)
Tindall et al., 2020 [70]	Subjects with elevated cardiovascular risk (*n* = 42) Males and females 30–65 years of age	Data/sample collected Fecal samples collected before diet and after diet phase Diet Six-weeks cross-over design using a walnut diet, a walnut- fatty-acid-matched diet or a oleic acid diet (all 48% carbohydrate, 35% fat, and 17% protein)	The following microbes were found in highest abundance after walnuts: *Roseburia*, *Eubacterium eligensgroup LachnospiraceaeUCG001*, *LachnospiraceaeUCG004* and *Leuconostocaceae*

### 4.3. Cell Culture Studies

Cell culture studies that have addressed mechanisms by which walnuts and their constituents affect cancer pathways are summarized in Table 3. Lee et al. [71] tested the effects of a walnut phenolic extract (WPE) and its bioactive compounds, including (+)-catechin, chlorogenic acid, EA and gallic acid, on CRC stem cells. CD133+/CD44+ cells were isolated from HCT116 cells and treated with WPE. Marked effects on stem cell survival included inhibition of cell differentiation and cell survival, and self-renewal capacity. A panel of stem cell markers were down-regulated, including CD133, CD44, DLK1 and Notch1, as well as Wnt signaling, a critical driver of CRC [72,73]. Importantly, the combined effects of WPE were stronger than the individual bioactives in the WPE [71]. The effects of an ethanolic extract from walnut kernels on oxidative damage in plasmid DNA and in normal human keratinocytes was examined [74]. Walnut extract protected DNA from tert-butyl hydroperoxide or UVC-induced irradiation damage. As part of a program to evaluate the effects of roasting conditions on bioactivity of various nuts, Schlormann et al. [72] tested chemo-preventive effects of walnuts under different roasting conditions. Focusing on this infrequently considered experimental variable, fermentation supernatants induced expression of detoxification genes (*CAT*, *GSTT2*), while inhibiting growth of LT97 colon adenoma cells, changes that were unaffected by moderate roasting [72]. Batirel et al. [73] investigated the effects of walnut oil (WO) on tumorigenesis and metastasis in OE19 human esophageal cancer cells. High-dose, short-term treatment with WO reduced cell viability and cancer metastatic potential, while exhibiting anti-carcinogenic effects via elevated necrosis and cell cycle arrest [73].

Choi et al. [75] investigated the effects of WPE on mitochondrial function in colon cancer stem cells (CSCs). CD133+/CD44+ HCT116 cells were isolated and treated with WPE. Combining mitochondrial functional assays (oxygen consumption and glycolysis) with gene expression profiling, they found that WPE treatment shifted the mitochondrial metabolism of CSCs towards aerobic glycolysis and oxidative pathways, suggesting a Warburg-like metabolic shift [75]. A total of 1168 up-regulated genes were classified into four functional clusters (metabolism, mitochondrion, apoptosis, and cancer pathways). Validation of metabolic and mitochondrial-related functional categories identified up-regulated genes related to mitochondrial activity [75]. Koh et al. [50] tested the effects of WPE on colitis-associated CRC in COLO205 cells. WPE caused a significant inhibition of IL-8 and Il-α expression, while attenuating TNF-α induced I-kB phosphorylation/degradation and NF-κB DNA binding [50,71]. Park et al. [76] explored walnut effects on *H. pylori* infection. WPE suppressed phosphorylation and nuclear translocation of STAT3 in *H. pylori*-infected RGM-1 gastric mucosal cells, and induced *SOCS1* and nuclear translocation of PPARγ [76]. WPE also inhibited the expression of c-Myc and IL-6/IL-6R signaling, indicating effects on distinct cancer pathways. The anti-cancer and antioxidant activities of phenolic compounds from black walnuts were tested using high-throughput screening in tumorigenic alveolar epithelial cells (A549) and non-tumorigenic lung fibroblasts (MRC-5) [77]. Of 16 phenolics tested for antioxidant activity, antioxidant response element (ARE) induction and anti-cancer activities, the most effective were penta-O-galloyl-β-d-glucose, epicatechin gallate, quercetin, (-)-epicatechin, rutin, quercetin 3-β-d-glucoside, gallic acid, (+)-catechin, ferulic acid and syringic acid [77].

Thirty-seven phenolic compounds (free, esterified and bound) present in walnut kernel were examined for antioxidant activities [39]. Radical scavenging activities were determined for these phenolic compounds. Additional transcriptomic profiling further ascertained how WPE can rescue gastric cells from *H. pylori* infection [78]. Multiple chemokine-related pathways were altered by WPEs, including an array of anti-inflammatory and anti-oxidative genes, providing further evidence for the potential impact of walnut extracts on modulating key signaling pathways that may ultimately contribute to the carcinogenic process present in *H. pylori*-infected gastric cells [78]. Nunez-Sanchez et al. [79] tested EA and its microbially-derived metabolites, the urolithins, at concentrations predicted in colon tissue following ingestion of EA-rich foods, based upon the established urolithin ‘metabotypes’ [52]. Urolithin combinations were administered to human CRC Caco-2 cells, and to primary tumor cells collected from a stage II CRC [79]. There was a decrease in both size and number of colonospheres in both cell lines and for either combination of urolithins. However, ADH activity, a biomarker of chemoresistance, was only reduced by the metabolite combination enriched in urolithin A, indicating a metabolite-specific response.

**Table 3:** Characteristics of cell culture studies (*n* = 11) included in a narrative review on the effects of walnut and walnut constituents on cancer risk and inflammation.

Author(s), Year, Reference	Cell Line/Compound Analyzed	Methods Used	Key Findings
Nunez-Sanchez et al., 2016 [79]	Caco-2 human colon cancer cells Primary colorectal cancer tumor cell established from a patient	Cells were treated with one of the following mixtures: Mixture 1 (MPhA) 85% Uro-A, 10% Uro-C, 5% EA mixture Mixture 2 (MPhB) 30% Uro-A, 50% IsoUro-A, 10% Uro-B, 5% Uro-C, 5% EA mixture	MphA inhibited the number and size of colonospheres and aldehyde dehydrogenase (ALDH) MPhB had modest effects on size and number of colonospheres, but no effect on ALDH
Wu et al., 2021 [39]	37 phenolic compounds from walnut kernels	Extraction of free, esterified, and bound forms of the 37 targeted walnut phenolics (WPs) using organic solvent/water solvent	51.5%–68.1% of WPs present in free forms 21.0%–38.0% of WPs present in bound forms 9.7%–18.7% WPs present in esterified forms Free forms of WPs had the highest radical scavenging activity
Schlormann et al., 2017 [72]	LT97 colon adenoma cells	LT97 cells were treated with fermentation supernatants (FS) from both raw and roasted walnuts	Increased mRNA expression of *CAT* and *GSTT2* and decreased *GPx1* Decreased cell growth Increased early apoptosis and induced activity of caspase-3
Lee et al., 2016 [71]	CD133+ CD44+ isolated from HCT116 human colon cancer cell line	CD133+ CD44+ HCT116 were treated with walnut phenolic extract (WPE) (0, 10, 20, and 40 ug/mL)	Inhibition of cell differentiation Downregulation of cancer stem cells (CSCs) Suppression of self-renewal of CSCs
Koh et al., 2019 [50]	COLO205 human colonic epithelial cell line	Cells were pretreated with WPE and stimulated with tumor necrosis factor (TNF-α)	Inhibition of IL-8 and IL-α expression in cells
Ho et al., 2020 [77]	Transfected liver cell line A546 (human alveolar epithelial cell line) MRC-5 (human lung fibroblast cell line)	Cells were treated with 16 phenolic compounds to test for antioxidant activity, anticancer activities, and antioxidant response element	Out of the 16 phenolic compounds, 9 showed antioxidant activity that was higher compared to control 2 phenolic compounds showed anti-proliferative activities against both A546 and MRC-5
Choi et al., 2019 [75]	CD133+ CD44+ HCT116 colon cancer cell line	Cells were treated with 40 ug/mL of WPE for 6 days	Metabolic and mitochondrial clusters found by RNA-Seq Treatment shifted the metabolic profile of cells towards glycolysis Increase in mitochondrial respiration Increased glucose uptake in cells
Batirel et al., 2018 [73]	OE19 esophageal adenocarcinoma cell line	Cells were treated with walnut oil (0–40 mg/mL)	Decreased cell viability Down-regulated NF-kB activity Induced cell-cycle arrest and necrosis at G0/G1 phase
Park et al., 2020 [76]	RGM-1gastric muscosal cells infected with *H. pylori*	Cells were treated with WPE (20 ug/mL)	Inhibited *H-pylori*-induced IL-6 Cox-2, c-Myc, and TNF-alpha expression Decreased STAT3 activation Induced SOCS1 expression that suppressed STAT3(tyr705) phosphorylation
Park et al., 2021 [78]	AGS human gastric adenocarcinoma cells infected with *H.pylori*	Cells were pretreated with WPE for 1 h and then stimulated with *H. pylori* for 48 h	Several newly identified genes were enriched by treatment with WPE Several genes that increased after *H. pylori* infection, were reduced after WPE treatment Several anti-inflammatory and anti-oxidative genes were altered by WPE treatment
Calcabrini et al., 2017 [74]	Keratinocyte cell line NCTC 2544	Antioxidant *Juglans regia* ethanolic extract (walnut kernel) DNA damage Induced by Thiol/Fe^3+^/O_2_ mixed function, tert-butyl hydroperoxide, or UVC radiations	Protection of oxidatively injured DNA

## 5. Walnut Effects on the Microbiome

Walnuts influence microbial community structure and the relative abundance of beneficial microbes. As shown in Figure 1, walnuts contain high levels of fiber that increase microbiome diversity and, in turn, increase the levels of SCFAs. SCFAs (acetate, butyrate, propionate) are important products of bacterial metabolism. Butyrate is a key energy source for colonocytes and has potent anti-cancer activity, primarily via its ability to inhibit histone deacetylases (HDACs), leading to changes in gene expression [80]. As butyrate levels increase in cancer cells, HDAC inhibition is associated with increased expression of key pro-apoptotic and cell-cycle regulatory genes, as well as downregulation of pro-inflammatory cytokines and NF-kB [80,81,82,83]. In addition, propionate, and acetate both play important roles in energy homeostasis. Acetate, the most abundant SCFA, can modulate bacterial growth and affect many metabolic processes, including lipogenesis [84].

The number of walnut-microbiome studies performed in animal models and in clinical studies is limited and they are reviewed in this section, summarized in Table 1 and Table 2.

### 5.1. Human Studies

The effects of daily walnut consumption (43 g/day) on the microbiome were tested [12] in a sub-study focused on lipid profiles in healthy volunteers [68]. The results of this randomized, prospective, cross-over study of 194 subjects showed that daily walnut ingestion over an 8-week period caused significant changes to microbial composition [12]. 16S rRNA sequencing showed that walnuts significantly altered gut microbiome composition and diversity. The abundance of *Ruminococcaceae* and *Bifidobacteria* was significantly increased by walnuts, whereas the abundance of *Clostridium* sp. Cluster XIVa species (*Blautia*; *Anaerostipes*) was reduced. Importantly, these investigators showed that walnut consumption enhanced the levels of probiotic- and butyrate-producing bacteria, providing key support for adding walnuts to a healthy diet [12].

A second and smaller, although scientifically rigorous study, was conducted in the U.S. by Holscher et al. [66]. Using a controlled-feeding, randomized crossover design, 18 study participants received isocaloric diets containing 0 or 42 g walnuts/day for two 3-week periods, with a 1-week run-in between diets. Interestingly, several of the microbial changes noted in this study were markedly different from the German study, perhaps reflecting distinct characteristics of these patient populations and their typical diets. Walnut consumption caused an increase in relative abundance of *Faecalibacterium*, *Clostridium*, *Dialister* and *Roseburia*, and reduced levels of *Ruminococcus*, *Dorea*, *Oscillospira* and *Bifidobacterium*. Importantly, the pro-inflammatory, microbially derived secondary bile acids, deoxycholic and lithocholic acids (DCA, LCA), were lowered by 25% and 45%, respectively, by walnuts. The differences observed in altered microbial composition between these two studies highlights some of the inherent challenges faced by investigators studying microbiome-diet interactions in diverse human populations.

Bacteria such as *Faecalibacterium prausnitzii* and *Roseburia intestinalis* ferment insoluble fiber into SCFAs including acetate, propionate, and butyrate [65]. SCFAs have been shown to suppress inflammation through activation of G-protein-coupled receptors on colonocytes lining the colon, triggering intracellular signaling pathways that suppress LPS-induced NF-kB activation, decrease pro-inflammatory mediators, increase anti-inflammatory mediators, and increase epithelial barrier function [85,86]. A recent study by Garcia-Mantrana et al. [69] shows that 3-day consumption of walnuts modulates the gut microbiota and increases the SCFA levels of healthy individuals, effects that depend upon the individuals’ microbiome. The study comprised 27 healthy subjects, and walnut consumption modulated the gut microbiota in an urolithin-metabolite-dependent manner. Gut microbiota profiling showed a significant increase in the species *Coprococcus* and *Anaerostipes*, producers of butyrate, and *Phascolarbacterium*, producer of acetate and propionate. The study also identified variability in the metabolism and bioactivity of polyphenols occurring between the different metabotypes [69]. 

Finally, a study from the Kris-Etherton lab [70] examined between-gut bacterial differences in a study population at increased risk of CV disease using a diet that replaced saturated fats with walnuts or vegetable oils. The study was a randomized, crossover, controlled-feeding trial with a two-week western diet run-in and 3 six-week isocaloric diets, including a walnut diet (57–99 g/day; 2.7% ALA). *Roseburia*, *Eubacterium eligensgroup*, *Lachnospiraceae*, *Lachnospiraceae* and *Leuconostocaceae* were found to be the most abundant taxa relative to the standard western diet. Interestingly, *Gordonibacter* was also enriched in the walnut diet [70].

### 5.2. Animal Studies

To gain further insight into the tumor protection afforded by walnuts in AOM-treated mice [45], Nakanishi and colleagues tested fecal samples using 16S rRNA sequencing and found that carcinogen exposure reduced the diversity and richness of the gut microbiome, especially in males, with lower variability and greater sensitivity to environmental changes [45]. The analysis of operational taxonomic units (OTUs) identified specific groups of bacteria associated with carcinogen exposure, walnut consumption and/or both variables. Correlation analysis identified specific OTU clades strongly associated with the presence of tumors [45]. The results indicate that walnuts afford partial protection to the colon against potent carcinogenic insult, due in part to the ability of walnuts to reshape the gut microbial community to one with anti-tumor properties. Interestingly, this study also established marked differences in microbiome signatures between male and female mice, wherein bacterial diversity increased more robustly in males after walnut consumption [45]. In a follow-up study, Chen et al. [51] evaluated walnut effects in these mice. Three enterotype-like clusters (E1, E2, and E3) were identified in TWD-fed mice treated with AOM and/or walnuts. E1, E2, and E3 were associated with AOM exposure, walnut consumption, and TWD diet, respectively. E2 and E3 showed distinct taxonomic and functional characteristics, while E1 represented an intermediate state. At the family level, E1 and E3 were both enriched with *Bacteroidaceae*, but driven by two different operational taxonomic units (OTU-2 for E1, OTU-4 for E3). E2 was overrepresented with *Porphyromonadaceae* and *Lachnospiraceae*, with OTU-3 (family *Porphyromonadaceae*) as the “driver” OTU for this cluster [51]. Functionally, E3 is overrepresented with genes of glycan biosynthesis and metabolism, xenobiotic metabolism, and lipid metabolism. E2 is enriched with genes associated with cell motility, replication and repair, and amino acid metabolism. Longitudinally, E2 represents the gut microbial status of early life in these mice. In comparison with E1 and E3, E2 is associated with a moderately lower tumor burden (*p* = 0.12). These results suggest that walnuts may reduce the risk of CRC in a western diet by altering the gut microbiota. These findings provide further evidence that CRC risk is potentially modifiable by diet via alterations to the microbiota [51].

Byerley et al. [52] also tested dietary walnut-induced changes to gut microbial communities in male Fischer 344 rats. Fiber, fat, and protein content were matched with corn oil, protein casein and a source of cellulose fiber. Intestinal samples were collected from the descending colon and 16S rRNA sequencing (V3-V4) was used for microbiome analysis. Walnut consumption resulted in significantly greater species diversity, increasing the abundance of *Firmicutes*, and reducing *Bacteroidetes*. Walnuts also enriched the microbiome for probiotic-type bacteria, including *Lactobacillus*, *Ruminococcaceae*, and *Roseburia* [52]. These investigators also found a significant reduction in *Bacteroides*, *Anaerotruncus* and the class *Alphaproteobacterial*.

## 6. Walnuts and Inflammation

Understanding how foods may directly affect aging processes associated with inflammation would help individuals to make optimal food choices, thereby reducing overall disease incidence. Figure 1 shows that walnut constituents contribute to decreased inflammation within the intestinal mucosa. This may relate, in part, to the microbial conversion of walnut-derived ellagitannins into the anti-inflammatory urolithins [87]. As noted earlier, walnuts also contain significant amounts of ALA, which can be converted to EPA and DHA, both possessing anti-inflammatory properties [88] (Figure 1). According to Grivennikov [89] inflammation in cancer may be related to its ability to cause mutations that ultimately lead to intestinal carcinogenesis. Inflammation leading to activated macrophages and neutrophils is a source of reactive oxygen species and reactive nitrogen species that are known to be highly mutagenic and DNA-damaging [90,91]. Chronic inflammation can also lead to the breakdown of the protective intestinal barrier, exposing the epithelium to various mutagens, leading to increased tumor initiation [89]. According to Grivennikov, inflammatory cytokines may play a significant role in the upregulation of oncogenes and the downregulation of tumor suppressor genes, resulting in part from epigenetic changes. Studies in animal models and cell culture systems have begun to illustrate the health benefits of walnuts, in part via effects on immune and inflammatory cells. Exploring the impact of dietary consumption of walnuts and their phytochemical composition could help substantiate their known health benefits and potentially uncover additional benefits yet to be recognized.

In a preclinical experimental model of ulcerative colitis (UC) using dextran sodium sulfate (DSS), the ability of EA to inhibit disease progression and reduce intestinal inflammation via down regulation of inflammatory mediators was tested [92]. Urolithin A and its potent synthetic analogue, UAS03, were also reported by Singh et al. [87] to mitigate DSS-induced intestinal inflammation, corresponding to reduced oxidative tissue damage and enhanced intestinal barrier function repair. Both urolithin A and UAS03 provided significant protection against acute and chronic colitis by causing a significant reduction in inflammatory mediators, up-regulation of the ligand-activated transcription factor, AhR, and enhanced barrier function via amplified expression of the tight junction protein, claudin 4 [87]. Since chronic inflammation is maintained through persistent activation and proliferation of CD4+ T cells, the effects of urolithin A were tested on the activation of these immune cells [93]. The resulting data provide compelling evidence for this ellagitannin metabolite acting as a natural therapeutic agent for inflammatory disorders. Koh et al. [50] demonstrated that WPE inhibits NF-κB signaling, associated with down-regulation of pro-inflammatory mediators, in acute and chronic experimental colitis models. It was further shown that WPE inhibited colitis-associated CRC in mice exposed to AOM followed by three cycles of 2% DSS for 5 days [50]. These findings not only highlight the therapeutic potential of walnuts in inflammatory disease but confirm its potential effectiveness in inflammation-associated cancer as well. Walnut oil (WO) was also shown to stabilize tight junction proteins and attenuate pro-inflammatory cytokines present in the inflamed mouse colon of DSS-treated mice [94]. WO improved the overall disease activity within the colon, while restoring normal ion transport and colonic wall permeability [94].

Nakanishi and colleagues recently evaluated dietary supplementation with walnuts on colonic mucosal injury induced by DSS [88]. Mice were fed TWD supplemented with walnuts (0–14% g/kg) for two weeks prior to DSS. Walnut supplementation showed significant protection to the colonic mucosa 10 days post-injury. Based on this protection by walnut against experimentally induced colitis, a follow-up study explored walnut effects on metabolites present in the colon [88]. A discovery-based metabolite-profiling analysis was performed on fecal and colonic samples two weeks post-walnut. Walnuts caused a significant increase in PUFAs, including DHA and 9-oxo-10(E),12(E)-octadecadienoic acid (9-oxoODA), as well as kynurenic acid in fecal samples. In colon, there was a significant increase in S-adenosylhomocysteine and betaine, important mediators of FA-β-oxidation. These results suggest that metabolic changes caused by walnuts may contribute to protection against DSS-induced inflammatory tissue injury [88]. Further studies are needed to confirm these findings and to better define the precise role of these metabolic changes on colonic inflammation.

Several clinical studies demonstrate that walnut supplementation, among other foods and nutrients, may alleviate the overall damaging effects of inflammation. The WAHA (Walnuts and Healthy Aging) study is a randomized clinical trial to determine if walnut consumption decreases inflammation in elderly subjects, monitored by systemic biomarkers [95]. Participants either abstaining from walnuts or consuming a diet with walnuts (15% energy; 30–60 g per day) were followed for two years. It was found that the walnut group had a significant reduction in a panel of inflammatory markers compared to baseline [95]. A study in Norway [96] provided evidence that dietary plant extracts, consisting of oregano, coffee, thyme, clove, and walnuts, may act synergistically to inhibit NF-kB activity. Combined plant extracts inhibited NF-kB activation in monocytes and in various organ sites, changes that were most pronounced with combined extract [96]. This synergistic effect supports the rationale for including dietary plant extracts as part of a healthy diet. In addition, the association of inflammatory processes with carcinogenic risk is well-known [97,98], further emphasizing the potential benefit of walnut consumption in managing long-term cancer risk.

Finally, a protective role for walnut fractions on development of gastric mucosal lesions, including gastritis, gastric ulcer, and gastric carcinoma, was tested [99]. Liu et al. [100] reported gastro-protective and cancer preventive effects of walnut constituents on alcohol-induced inflammation, with fewer gastric lesions and decreased gastric inflammation associated with decreased inflammatory cytokines. Park et al. [101] tested the anti-inflammatory and anti-tumorigenic benefit of walnuts in an *H. pylori* gastric cancer model. Mice infected with *H. pylori* and fed a high-salt diet to promote gastric cancer were supplemented with walnut for nine months. Walnuts caused a significant reduction in gastric cancer frequency with markedly reduced levels of PGE_2_ and COX-2, important pro-inflammatory mediators that play a key role in tumor promotion [100,101].

## 7. Limitations and Future Directions

This review was compiled from 33 individual studies, including clinical trials, preclinical models, and cell culture systems, providing a general overview of our current understanding of the bioactive properties of walnut and its constituents. While this review summarizes a broad range of studies focused on colorectal health, there are certain limitations that should be considered. For example, a key limitation to animal studies is their typically short-term nature and the often-undefined composition of the resident gut microbiome. Notably, some mouse lines lack the capacity to form urolithins from ellagic acid [45]). Thus, using mouse cancer models with a defined (or humanized) microbiome [102] would be an important future direction that could applied for studying walnuts, phytochemicals, and their cancer preventive properties. Several clinical trials have made significant inroads regarding the health benefits of walnuts, particularly with respect to cardiovascular and cognitive effects [3,26,70]. Despite these reported benefits, however, limited trial size with respect to the colon [66,69] can also be confounded by inherent patient heterogeneity, particularly with respect to gut microbiome composition, which further complicates data interpretation. This is especially the case regarding the metabolism of walnut-derived ellagitannins. In addition, investigator control over dietary intake patterns in clinical studies can present significant challenges. While combining BFFQ and diet recall surveys to evaluate dietary habits and patient compliance are clearly helpful, it is difficult to ensure full patient compliance, especially with respect to avoiding specific food groups that may confound the results.

It is also interesting to note that that there are many other plant-based biocompounds that have been studied for their anti-cancer properties, acting through both anti-inflammatory and antioxidant effects, such as withaferin A, found in *Withania somnifera* and *Cleome* species [103,104].

## 8. Conclusions

Foods rich in anti-inflammatory and antioxidant compounds show promise for prevention of chronic inflammatory diseases and cancer, including CD and related disorders. Walnuts contain an array of natural constituents with additive and/or synergistic properties contributing to improved health. Walnuts are a rich source of ALA, tocopherols, antioxidant polyphenols (including ellagitannins) and fiber. There is also new evidence that walnuts have prebiotic potential, promoting growth of beneficial gut bacteria. Studies supporting microbiome- and metabolite-modifying potential include preclinical studies and promising clinical trials, including work ongoing in the laboratory. Mediated both directly and indirectly via actions on the microbiome, many beneficial properties of walnuts are associated with a range of anti-inflammatory properties. Among the most potent constituents of walnuts are the ellagitannins, primarily pedunculagin. After ingestion, the ellagitannins are converted to EA, a non-flavonoid polyphenolic that is subsequently metabolized by gut microbiota to bioactive, anti-inflammatory urolithins. This review has summarized the current knowledge regarding walnut effects on human health and suggests that walnut incorporation into the diet may offer a simple, cost-effective strategy for providing wide-ranging health benefits.
